# Curved TVs improved watching experience when display curvature radii approached viewing distances: Effects of display curvature radius, viewing distance, and lateral viewing position on TV watching experience

**DOI:** 10.1371/journal.pone.0228437

**Published:** 2020-02-06

**Authors:** Sungryul Park, Gyouhyung Kyung, Jihhyeon Yi, Donghee Choi, Songil Lee

**Affiliations:** 1 Department of Human Factors Engineering, UNIST, Ulsan, Republic of Korea; 2 Division of Media, Culture and Design Technology, Hanyang University, Ansan, Republic of Korea; Fondazione Ugo Bordoni, ITALY

## Abstract

Although watching TV often involves multiple viewing distances and viewers, less attention has been paid to the effects of display curvature radius, viewing distance, and lateral viewing position on TV watching experience. This study examined the effects of four display curvature radii (2300R, 4000R, 6000R, and flat), two viewing distances (2.3 m and 4 m), and five lateral viewing positions (P_1_-P_5_; 0, 35, 70, 105, and 140 cm off-center) on seven TV watching experience elements (spatial presence, engagement, ecological validity, negative effects, visual comfort, image quality, and user satisfaction). Fifty-six individuals (14 per display curvature radius) were seated in pairs to watch videos, each time at a different viewing position (2 viewing distances × 5 paired lateral viewing positions). The spatial presence and engagement increased when display curvature radius approached a viewing distance and lateral viewing position approached P_1_, with 4000R-4m-P_1_ (display curvature radius-viewing distance-lateral viewing position) providing the best results. Lateral viewing position alone significantly affected five TV watching experience elements; the spatial presence and engagement decreased at P_3_-P_5_, and ecological validity, image quality, and user satisfaction decreased at P_4_-P_5_. However, display curvature radius alone did not appreciably affect TV watching experience, and viewing distance alone significantly affected visual comfort only, with a 4-m viewing distance increasing visual comfort. This study demonstrated that effective display curvature radii for watching TV are viewing distance-dependent, and less off-center lateral viewing positions (P_1_-P_2_) are recommended for TV watching experience. Finally, among the TV watching experience elements, engagement explained user satisfaction to the greatest degree.

## Introduction

Media form factors [1] including display size, viewing distance, and image quality [2] influence watching experience by, for example, affecting geometric distortion and brilliance [3]. Display curvature radius, as a new media form factor, can increase presence [4], visual comfort [5], image quality [4], preference [6], and legibility [7], and reduce visual fatigue [8] and perceptual distortion [9]; however, it can also induce negative shape aftereffects [10, 11] and longer visual processing times [12]. Herein, visual comfort is defined as the subjective impression of comfort caused by visual stimuli [13], and image quality, as an important evaluation factor for TV watching experience [14], is subjectively determined through a comparison of the displayed image and the viewer’s image impression [15].

Unlike other curved display products (e.g. monitors, smartphones), curved TVs often involve multiple viewing distances for multiple viewers. When multiple viewers are involved, only one viewer can be centered in front of the TV, while other viewers should sit off-center. Yet, the comprehensive effects of display curvature radius, viewing distance, and lateral viewing position on TV watching experience remain largely unknown. TV watching experience comprises diverse elements. In previous studies on TV watching experience, presence [1, 16–19], visual comfort [20–26], image quality [17, 27, 28], satisfaction [26], visual fatigue [26, 29–31], motion sickness [16], empirical 3-dimensional (3D) image distortion [21], and emotional reactions [28] were considered. User satisfaction is used to explain the overall quality of experience with visual display products.

Display curvature radius, viewing distance, and lateral viewing position can influence TV watching experience as these factors affect the display field of view and viewing angles across the screen. For a given display size, if the display curvature radius approaches the viewing distance, the display field of view increases, while the variation in the viewing distances and viewing angles is less across the screen. Herein, the viewing angle refers to the angle between a horizontal line of sight and a normal line at a fixation point on the display surface. If the viewing distance decreases (if a viewer sits closer to the display), the display field of view increases. If the viewing position is more off-center, the viewing distance and viewing angle vary more across the screen and increase with respect to the center of display surface. A wider display field of view increases presence as a wider screen image occupies the viewer’s visual field to a greater degree [32]. Providing less varying viewing distances across the screen can reduce visual discomfort by reducing accommodation-vergence activities required for clear vision, whereas potential visual fatigue due to a prolonged visual task at similar focal distances [33] appears to be diminished by the aforementioned benefit [7]. Less varying viewing angles across the screen can enhance image quality, as it reduces the perceived distortion of an image displayed at the edge of the display [9]. A wider viewing angle can negatively affect image quality and visual comfort because the perceived image distortion increases with increasing viewing angles [34]. Thus, the display curvature radius, viewing distance, and lateral viewing position can affect TV watching experience and ultimately user satisfaction.

Viewing distance is generally determined by display size and image quality. When the viewing angle increases, the visual stimuli on the display become distorted [35]. Although presence increases as viewing distance decreases, it can suffer at excessively short viewing distances [36, 37]. Studies on non-high definition (HD) flat TVs have involved viewing distances of 2–14 W [38, 39] and 5 H [40], where W and H respectively represent display width and height, whereas HD TV studies have used shorter viewing distances (3–4 W or 0.8–6 H) [27, 41–46]. No study, however, has addressed the interactive effect of viewing distance and display curvature radius on TV watching experience.

Similarly, lateral deviations in viewing position (or increases in viewing angle) can affect TV watching experience. Although images viewed at an angle experience trapezoidal distortions [47], non-central viewing positions are sometimes inevitable, especially in multi-viewer conditions. Typical viewing angles in such conditions range between ±60° [48] with a mean viewing angle of 23.3° [49]. Indeed, 73% of South Korean households in 2015 [50] and 70% of US households in 2012 [51] had more than one member, indicating watching TV together is common in most households. However, the degree to which viewing angle (or lateral viewing position) affects TV watching experience remains largely unknown.

Valid TV watching experience studies should carefully address in-context settings [52]. Some previous studies on flat or curved TVs have used restrictive settings [involving single viewing distances [11, 53], centralized viewers [53, 54], or exclusively static images [55, 56]. Further research incorporating dynamic images is thus required to examine the comprehensive effects of display curvature radius, viewing distance, and lateral viewing position on TV watching experience.

Thus far, many TV watching experience elements have been considered: presence [16, 19], visual comfort [24, 26], image quality [17, 28], satisfaction [26], visual fatigue [26, 29], motion sickness [16, 57], image distortion [21], and emotional reactions [28]. The spatial presence felt by a display user can act as a predictor for user satisfaction [58]. Image quality and video quality, as the main elements of quality of experience [41, 42], also accounts for user satisfaction [59] and customer satisfaction [60]. A previous study on the development of an engagement scale for TV watching proposed a conceptual model that explains the effect of media and content characteristics on presence and the effect of presence on post-satisfaction [61]. However, no widely known study has considered major TV watching experience elements simultaneously. Moreover, it is largely unknown as to which TV watching experience elements can effectively explain user satisfaction associated with TV watching.

**Fig 1:**
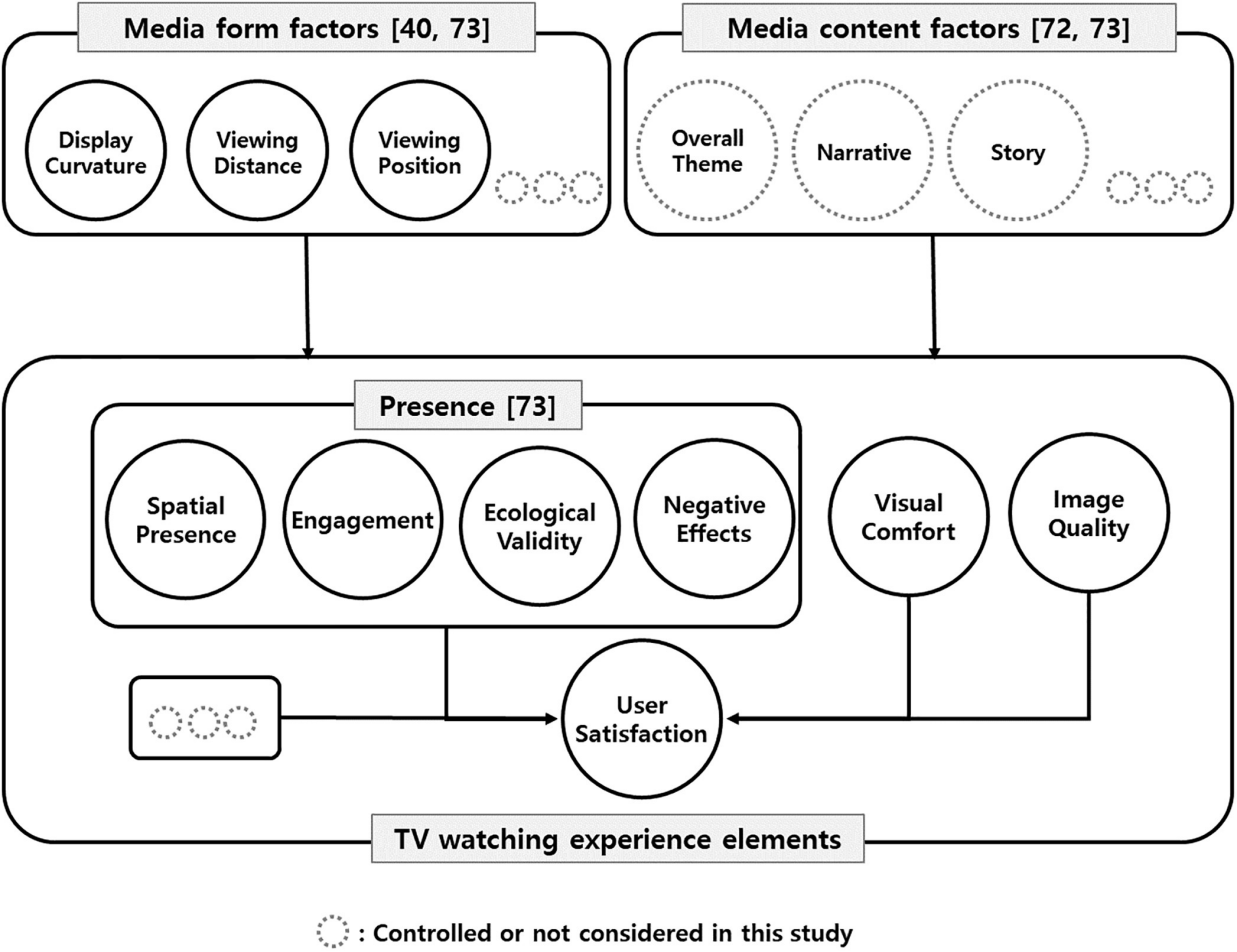
Hypothetical model for causal relationships between media form/content factors and TV watching experience, and between other TV watching experience elements and user satisfaction.

Thus, this study aimed to generate ergonomic guidelines for three major media form factors (display curvature radius, viewing distance, and lateral viewing position) to improve the overall TV watching experience and particular TV watching experience elements. These three media form factors and seven major TV watching experience elements (spatial presence, engagement, ecological validity, negative effects, visual comfort, image quality, and user satisfaction) were considered to examine 1) the main and interactive effects of these three media form factors on each TV watching experience element and 2) the relative importance of each TV watching experience element in explaining user satisfaction (Fig 1).

## Materials and methods

### Design and subjects

This study recruited 56 college students (Table 1), selected based on the following criteria: 1) normal or corrected–to–normal visual acuity ≥ 0.8 (20/25 in Snellen notation) for both eyes [62] determined using the Han Chun Suk visual acuity chart [63], 2) non-color deficiency determined using the Ishihara color blindness test [64], 3) no vision-related illnesses in the last six months, and 4) non-glasses or contact lens wearers. The study protocol was approved by the Ulsan National Institute of Science and Technology (UNIST) Institutional Review Board (IRB). All the participants provided written informed consent, which the local IRB approved, and were compensated for their time.

**Table 1:** Participant characteristics: Age and visual acuity.

Display curvature radius	# of participants (male, female)	Mean (SD) age	Mean (SD) visual acuity	
			Left	Right
**2300R**	14 (6, 8)	22.4 (1.1)	1.1 (0.3)	1.1 (0.2)
**4000R**	14 (4, 10)	20.9 (1.9)	1.0 (0.2)	1.0 (0.2)
**6000R**	14 (8, 6)	20.1 (1.4)	1.0 (0.2)	1.0 (0.2)
**Flat**	14 (2, 12)	20.1 (1.4)	1.0 (0.2)	1.0 (0.2)

### Experimental settings and procedures

**Fig 2:**
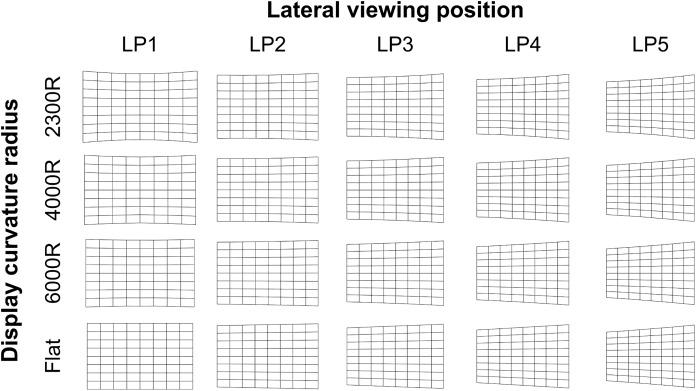
Grid images on differently curved surfaces viewed at different viewing positions.

Laboratory experiments were conducted with external lights blocked using black curtains and black cloth covering the TV stand and walls to minimize color and light reflection. Each experimental TV mock-up consisted of projection film (EXZEN, Korea) attached to the front surface of a 55" (1218 mm × 685 mm; 16:9 aspect ratio) custom Styrofoam panel, and was placed on a stand (320 mm high) elevating the display center 648 mm from the floor. The gain of the projection films attached to the curved screen surfaces was 1.0. Display size is defined as the length of a straight or curved diagonal along the screen surface. Each particular combination of display curvature radius and lateral viewing position changed the actual viewing distance, viewing angle, and display field of view (Table 2), and provided on-screen images from different perspectives (Fig 2).

**Table 2:** Actual viewing distance, viewing angle, and field of view according to display curvature radius, viewing distance, and lateral viewing position.

Viewing distance (m)		Display curvature radius (mm)	Lateral viewing position				
			P_1_	P_2_	P_3_	P_4_	P_5_
**2.3**	**Actual viewing distance (m)**	-	2.3	2.3	2.4	2.5	2.7
	**Viewing angle (°; display center)**	-	0.0	8.7	17.0	25.0	31.0
	**Viewing angle (°; across horizontal surface)**	2300R	0.0–0.0	8.0–8.7	15.2–17.7	21.5–26.6	26.9–34.9
		4000R	0.0–6.3	2.3–14.2	11.1–21.3	19.9–27.6	28.2–32.9
		6000R	0.0–9.2	0.7–17.1	8.1–24.2	16.8–30.4	25.0–35.7
		Flat	0.0–14.8	6.4–22.6	2.3–29.6	10.9–35.8	19.0–41.1
	**Field of view (°)**	2300R	30.3	29.7	27.9	25.2	22.3
		4000R	30.1	29.5	27.7	25.2	22.3
		6000R	30.0	29.4	27.6	25.1	22.3
		Flat	29.7	29.1	27.4	24.9	22.2
**4.0**	**Actual viewing distance (m)**	-	4.0	4.0	4.1	4.1	4.2
	**Viewing angle (°; display center)**	-	0.0	5.0	9.9	15.0	19.0
	**Viewing angle (°; across horizontal surface)**	2300R	0.0–6.4	1.5–11.5	3.2–16.6	7.7–21.7	11.9–26.6
		4000R	0.0–0.0	4.9–5.0	9.6–10.1	14.0–15.1	18.2–20.1
		6000R	0.0–2.9	2.1–7.8	7.1–12.4	12.2–16.9	17.1–21.0
		Flat	0.0–8.7	3.7–13.5	1.3–18.1	6.3–22.5	11.2–26.7
	**Field of view (°)**	2300R	17.5	17.3	16.9	16.3	15.5
		4000R	17.4	17.3	16.9	16.3	15.6
		6000R	17.4	17.3	16.9	16.3	15.5
		Flat	17.3	17.2	16.8	16.2	15.5

Each Styrofoam panel had a particular display curvature radius (2.3 m (2300R), 4 m (4000R), 6 m (6000R), and flat). A 5.1 channel speaker system (BR-5100T2, Britz, Korea) with one subwoofer on the left of the stand, one speaker on the right, and one speaker in each of the room corners was used. Video images were projected on each projection film by using a beam projector (EB-4950WU, Epson) with a wide ultra-extended graphics array (WUXGA; 1920 × 1200) resolution and a temporal frequency of 60 Hz. To correct the distortion of the image projected on the flat and curved screens, a 9(W) × 9(H) rectangular grid was displayed on the screen surface. Then, grid intersections were positioned to reference points by using Desktop Warpalizer^®^ (UniVisual Technologies, Sweden).

**Fig 3:**
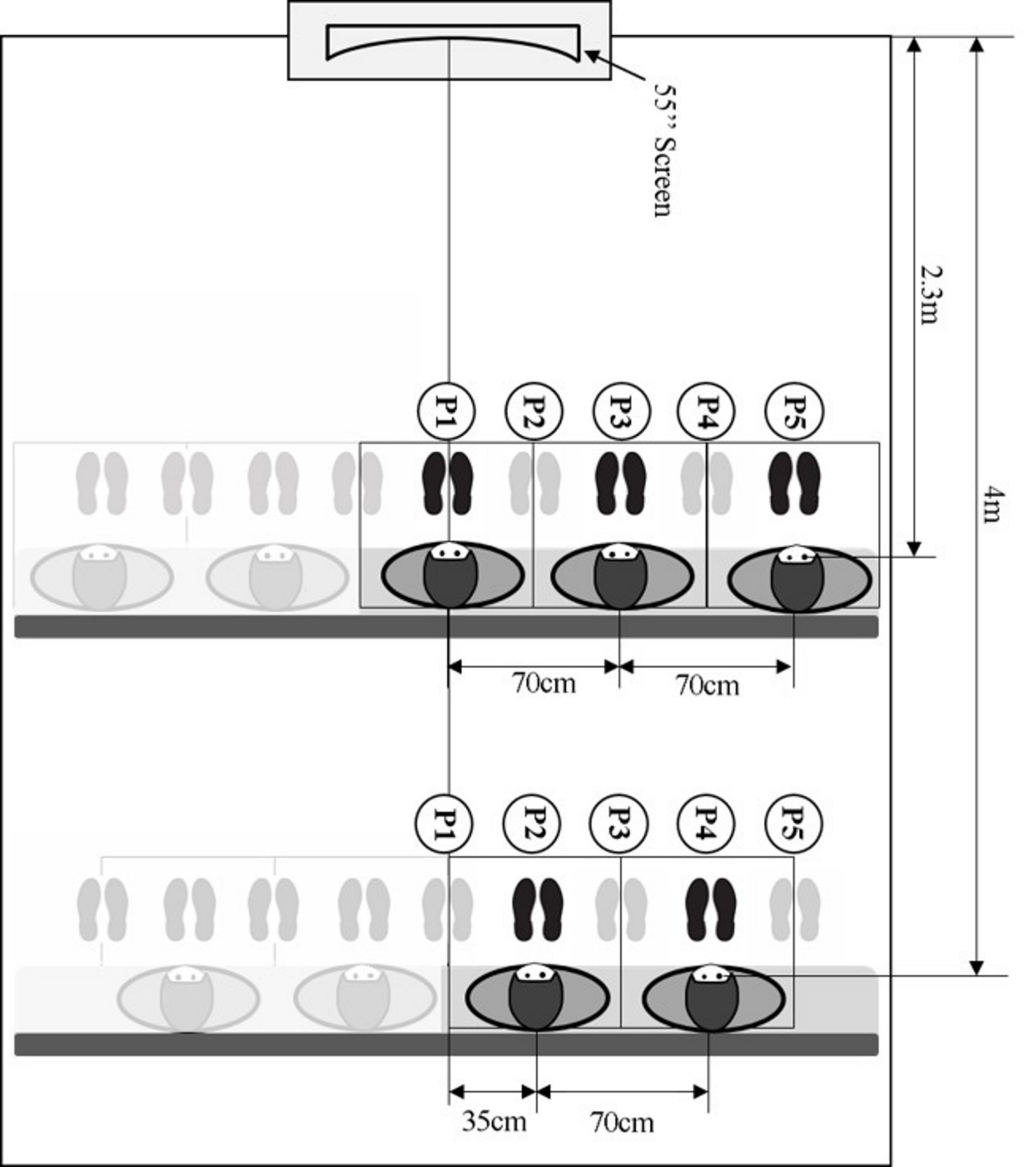
Viewing distances and lateral viewing positions (Five paired lateral viewing positions, P_1_-P_3_, P_2_-P_4_, P_3_-P_5_, P_4_-P_2_, and P_5_-P_1_, were used for viewing distances of 2.3 m and 4 m).

Seven random pairs of individuals were assigned to one display curvature radius. Two viewers were seated together in the randomly selected paired lateral viewing positions on a sofa (width × depth × height: 250 × 60 × 45 cm). A total of five pairs of right-side lateral viewing positions were considered, assuming viewers sat with lateral symmetry (Fig 3). With one exception (P_5_-P_1_), two viewers sat 70 cm apart [65]. The first viewing distance for the current paired viewers was the second viewing distance for the previous paired viewers.

**Fig 4:**
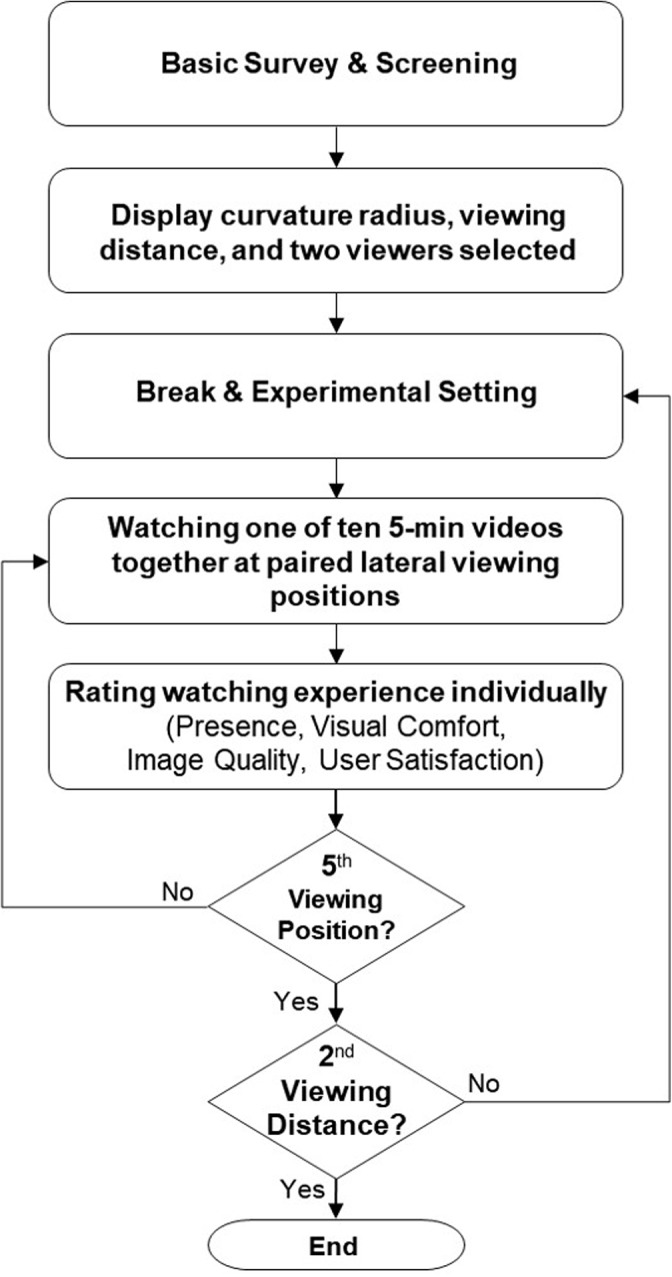
Experimental procedure.

Previous studies on presence, visual comfort, image quality, and user satisfaction used a wide range of viewing durations, from 90 s to 4 h [17, 25–27, 31, 40, 66–70]. A randomly selected video from ten 5 min videos was used for each combination of viewing distance × lateral viewing position. Each video consisted of five 1 min clips (motorcycling, car chases, roller coaster riding, combat flying, and scenic flying). TV watching experience was rated after each video was watched (Fig 4).

### Independent variables

Three independent variables were investigated. The display curvature radius varied between subjects at four levels: 2300R (providing a 30° ‘effective’ field of view [71] at a 4 m viewing distance), 4000R and 6000R (adopted in commercialized TV models: UN55JU7550F, Samsung, Korea; and 105UC9, LG, Korea), and flat (the control treatment). All participants used two viewing distances [2.3 m and 4 m, respectively equivalent to 1.9 display width (W) or 3.4 display height (H) and 3.3 W or 5.8 H] and five lateral viewing positions [P_1_ (centered in front of the TV), P_2_ (35 cm to the right of P_1_), P_3_ (70 cm off-center), P_4_ (105 cm off-center), and P_5_ (140 cm off-center)]. A wide range of viewing distances, 2–14 W and 0.8–7 H, have been used in previous studies, which will be reviewed later in the study (see Table 5). Five pairs of lateral viewing positions (P_1_-P_3_, P_2_-P_4_, P_3_-P_5_, P_4_-P_2_, and P_5_-P_1_) were used in random order, with the second individual 70 cm to the right of the first in all configurations but P_4_-P_2_, and P_5_-P_1_ (Fig 3).

### Dependent variables

Seven dependent variables were used to assess TV watching experience: spatial presence, engagement, ecological validity, negative effects, visual comfort, image quality, and user satisfaction. Spatial presence is defined as “a binary experience, during which perceived self-location and, in most cases, perceived action possibilities are connected to a mediated spatial environment, and mental capacities are bound by the mediated environment instead of reality [72].” Engagement is defined as “a measure of a user’s involvement and interest in the content of the displayed environment, and their general enjoyment of the media experience [73].” Ecological validity is defined as “a tendency to perceive the mediated environment as lifelike and real [73].” Negative effects describe adverse physiological reactions, including dizziness, nausea, headache, and eyestrain [73].

The first four variables are sub-concepts of presence [73], and were assessed using 13 items selected from the Independent Television Commission-Sense of Presence Inventory (ITC-SOPI): three regarding spatial presence (Q7, 9, 18), three regarding engagement (Q2, 8, 16), three regarding ecological validity (Q5, 11, 27), and four regarding negative effects (Q14, 21, 26, 37). Each item was rated on a 5-point Likert scale (0: strongly disagree, 1: disagree, 2: neutral, 3: agree, 4: strongly agree), and the mean item values of each sub-concept were used in statistical analyses. Visual comfort, image quality, and user satisfaction were respectively rated on a 100 mm visual analogue scale (VAS) (0: Very uncomfortable, 100: Very comfortable), a 5-point scale (bad, poor, fair, good, and excellent), and a 100 mm VAS (0: Very dissatisfied, 100: Very satisfied).

### Statistical analysis

A three-way mixed factorial analysis of variance (ANOVA; [74]) was used to examine the main and interaction effects of display curvature radius (four-level between-subjects variable), viewing distance (two-level within-subjects variable), and lateral viewing position (five-level within-subjects variable) on each of the seven dependent variables described in the previous subsection. When an effect was observed to be significant, the Tukey’s honestly significant difference (HSD) test was conducted [75]. For the Likert scale and image quality data, the distances between any two adjacent points along the rating scale were assumed to be equal, and all these data were treated as interval-like. The effect size was interpreted as low, medium, or high when the partial η^2^ was 0.01, 0.06, or 0.14, respectively [76, 77]. A stepwise multiple linear regression analysis was performed to examine the degree to which user satisfaction variability (satisfaction associated with watching TV) was accounted for by the other six TV watching experience elements. A p-value of 0.1 (for each predictor to enter or leave the model) was applied as a threshold during the construction of the stepwise multiple linear regression model [78, 79]. All statistical analyses were performed using JMP^™^ (v12, SAS Institute Inc., NC, USA), with a significance threshold of p < 0.05.

## Results

This section presents the results of the ANOVA examining the effects of three media form factors (display curvature radius, viewing distance, and lateral viewing position) on seven TV watching experience elements (presence, visual comfort, image quality, and user satisfaction; Table 3 with *p* values, *F* ratios, and effect sizes [partial η^2^]). In addition, it describes the regression model developed to determine the relative importance of each TV watching experience element in explaining user satisfaction.

**Table 3:** P-values for main and interaction effects of three media form factors (display curvature radius, viewing distance, and lateral viewing position) on seven TV watching experience elements (spatial presence, engagement, ecological validity, negative effects, visual comfort, image quality, and user satisfaction).

Effects	Presence				Visual comfort	Image quality	User satisfaction
	Spatial presence	Engagement	Ecological validity	Negative effects			
**Display curvature radius**	.52 (F_3, 52_ = .76; .04)	.93 (F_3, 52_ = .45; .03)	.39 (F_3, 52_ = 1.02; .06)	.025* (F_3, 52_ = 3.37; .03)	.98 (F_3, 52_ = .05; .003)	.85 (F_3, 52_ = .26; .02)	.99 (F_3, 52_ = .03; .002)
**Viewing distance**	.29 (F_1, 52_ = 1.13; .02)	.26 (F_1, 52_ = 1.28; .02)	.21 (F_1, 52_ = 1.59; .03)	.067 (F_1, 52_ = 3.51; .07)	.035* (F_1, 52_ = 4.67; .08)	.18 (F_1, 52_ = 1.89; .04)	.39 (F_1, 52_ = 1.68; .03)
**Lateral viewing position**	<.0001* (F_4, 208_ = 20.08; .28)	<.0001* (F_4, 208_ = 13.19; .20)	<.0001* (F_4, 208_ = 9.39; .15)	.071 (F_4, 208_ = 2.19; .04)	.34 (F_4, 208_ = 1.15; .02)	.0001* (F_4, 208_ = 4.88; .09)	<.0001* (F_4, 208_ = 8.35; .14)
**Display curvature radius × viewing distance**	.086 (F_3, 52_ = 2.32; .12)	.11 (F_3, 52_ = 2.12; .11)	.087 (F_3, 52_ = 2.31; .12)	.27 (F_3, 52_ = 1.36; .07)	.11 (F_3, 52_ = 2.08; .11)	.84 (F_3, 52_ = .28; .02)	.71 (F_3, 52_ = .45; .03)
**Display curvature radius × lateral viewing position**	.28 (F_12, 208_ = 1.20; .07)	.55 (F_12, 208_ = .90; .05)	.20 (F_12, 208_ = 1.34; .07)	.84 (F_12, 208_ = .60; .03)	.58 (F_12, 208_ = .87; .05)	.32 (F_12, 208_ = 1.15; .06)	.09 (F_12, 208_ = 1.62; .09)
**Viewing distance × lateral viewing position**	.24 (F_4, 208_ = 1.38; .026)	.20 (F_4, 208_ = 1.50; .028)	.031* (F_4, 208_ = 2.72; .050)	.67 (F_4, 208_ = .59; .011)	.25 (F_4, 208_ = 1.36; .025)	.26 (F_4, 208_ = 1.33; .025)	.41 (F_4, 208_ = .99; .026)
**Display curvature radius × viewing distance × lateral viewing position**	.004* (F_12, 208_ = 2.50; .13)	.022* (F_12, 208_ = 2.05; .11)	.065 (F_12, 208_ = 1.72; .09)	.082 (F_12, 208_ = 1.64; .09)	.83 (F_12, 208_ = .61; .03)	.72 (F_12, 208_ = .74; .04)	.14 (F_12, 208_ = 1.47; .08)

*p-values < .05; F-ratio with corresponding degrees of freedom and partial η^2^ denoted in parentheses

### Overview

The interaction effect of display curvature radius × viewing distance × latera viewing position was significant with a medium effect size (*partial η*^*2*^ *= 0*.*13*) for spatial presence and engagement. Spatial presence and engagement increased when display curvature radius approached viewing distance and lateral viewing position was less off-center, with 4000R-4m-P_1_ (display curvature radius-viewing distance-lateral viewing position) being the best combination. The interaction effect of viewing distance × lateral viewing position was significant with a small effect size (*partial η*^*2*^ *= 0*.*05*) for ecological validity; ecological validity decreased at a 2.3 m viewing distance and at more off-center lateral viewing position s. Display curvature radius alone did not appreciably affect TV watching experience. Viewing distance alone significantly affected visual comfort only, with a medium effect size (*partial η*^*2*^ *= 0*.*08*); visual comfort decreased at 2.3 m viewing distance. In contrast, lateral viewing position alone significantly affected five TV watching experience elements, with medium-to-large effect sizes (*partial η*^*2*^ *= 0*.*09–0*.*28*). Spatial presence and engagement decreased significantly at a lateral viewing position ≥ 70 cm, whereas ecological validity, image quality, and user satisfaction decreased significantly at a lateral viewing position ≥ 105 cm. Finally, six TV watching experience elements accounted for 67% of user satisfaction variability, with engagement being the most influential.

### Interaction effects of display curvature radius × viewing distance × lateral viewing position

**Fig 5:**
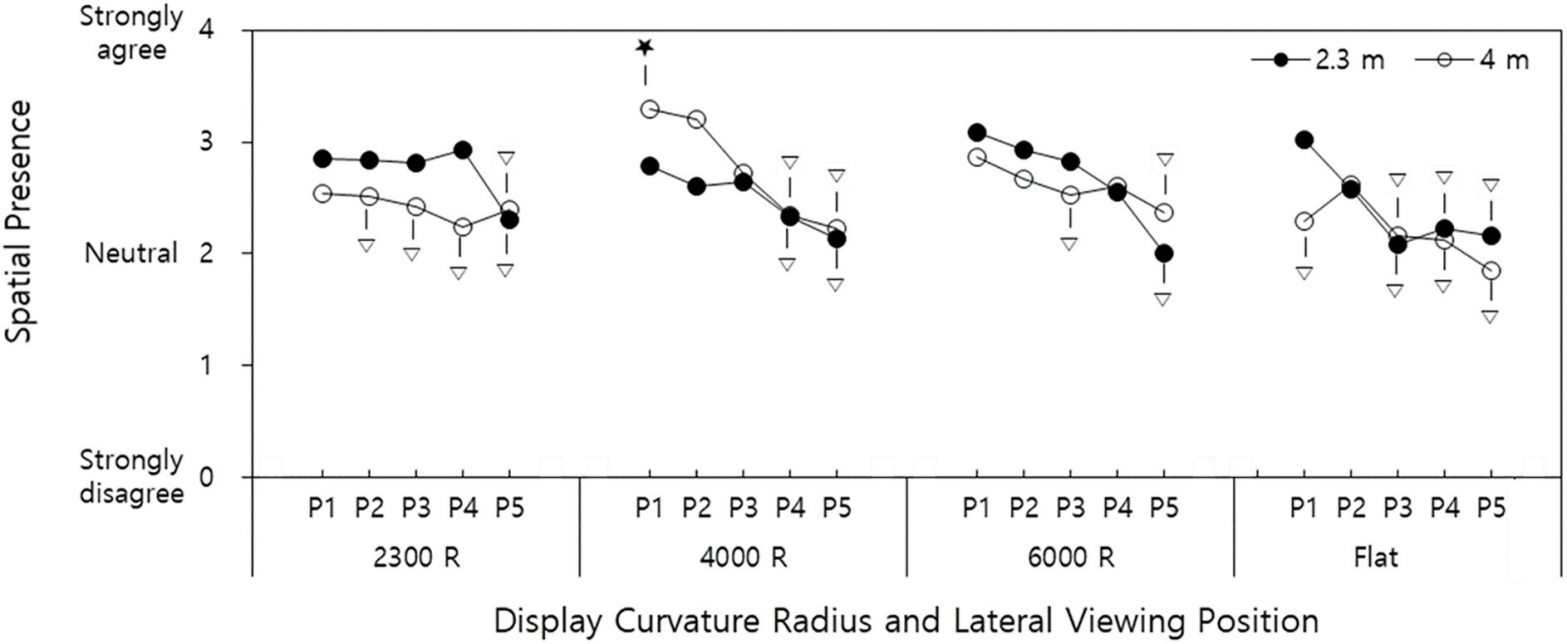
Interactive effects of display curvature radius × viewing distance × lateral viewing position on spatial presence (P_1_: Central position, P_5_: Rightmost position (140 cm off-center). Among the treatments belonging to Group A according to Tukey’s HSD test, the treatment with the highest mean spatial presence denoted as ★. Treatments not belonging to Group A denoted as ▽. Treatments without ▽ belong to Group A with the treatment with ★. Range of SEs: 0.03–0.13).

The interaction effect of display curvature radius × viewing distance × lateral viewing position was significant for spatial presence (*p = 0*.*004*). Twenty of the 40 treatments were in the same group (A) with 4000R-4m-P_1_ (display curvature radius-viewing distance-lateral viewing position), which provided the highest mean (SD) spatial presence of 3.3 (0.5) (Fig 5).

**Fig 6:**
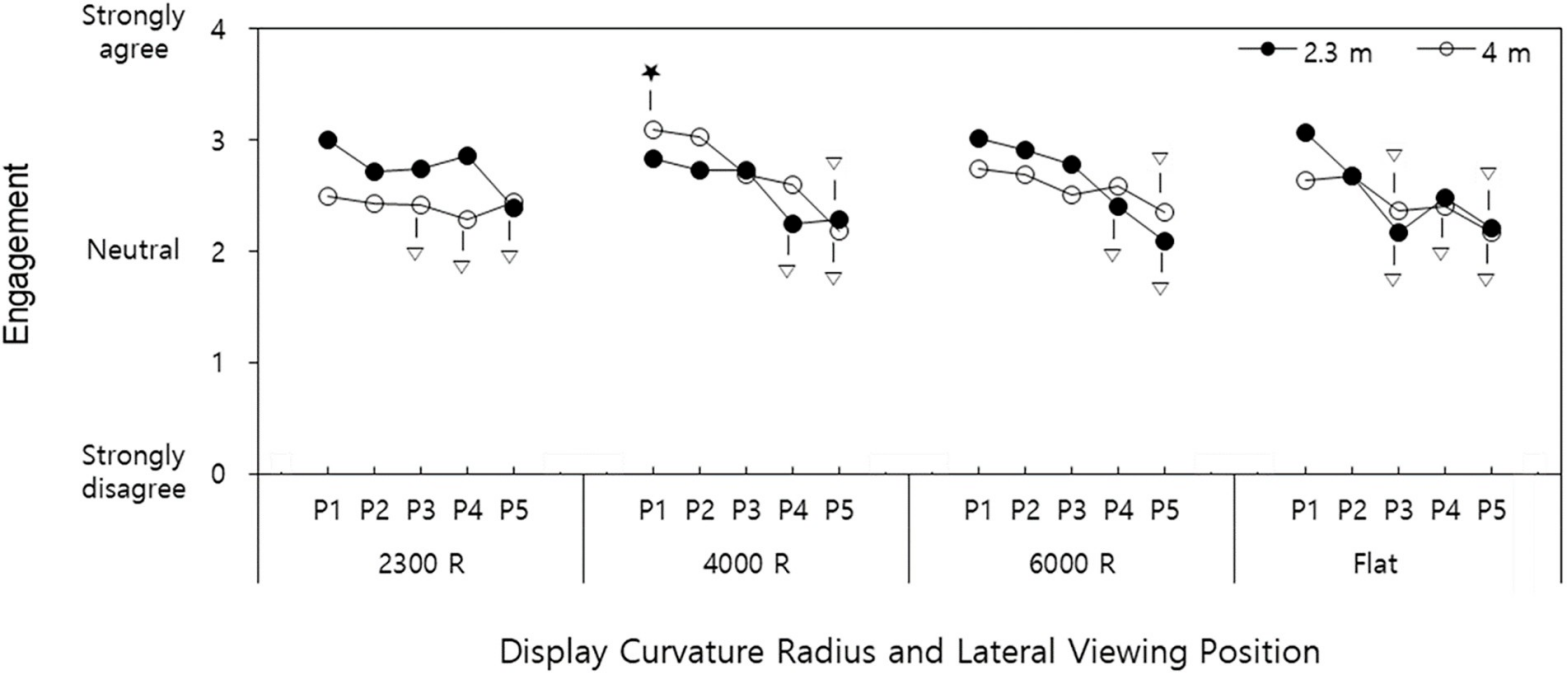
Interactive effects of display curvature radius × viewing distance × lateral viewing position on engagement (P_1_: Central position, P_5_: Rightmost position (140 cm off-center). Among the treatments belonging to Group A according to Tukey’s HSD test, the treatment with the highest mean engagement denoted as ★. Treatments not belonging to Group A denoted as ▽. Treatments without ▽ belong to Group A with the treatment with ★. Range of SEs: 0.05–0.11).

The interaction effect of display curvature radius × viewing distance × lateral viewing position was significant for engagement (*p* = 0.022). Of 40 treatments, 25 were in the same group (A) with 4000R-4m-P_1_ (display curvature radius-viewing distance-lateral viewing position), which provided the highest mean (SD) engagement of 3.1 (0.6) (Fig 6).

### Interaction effects of viewing distance × lateral viewing position

**Fig 7:**
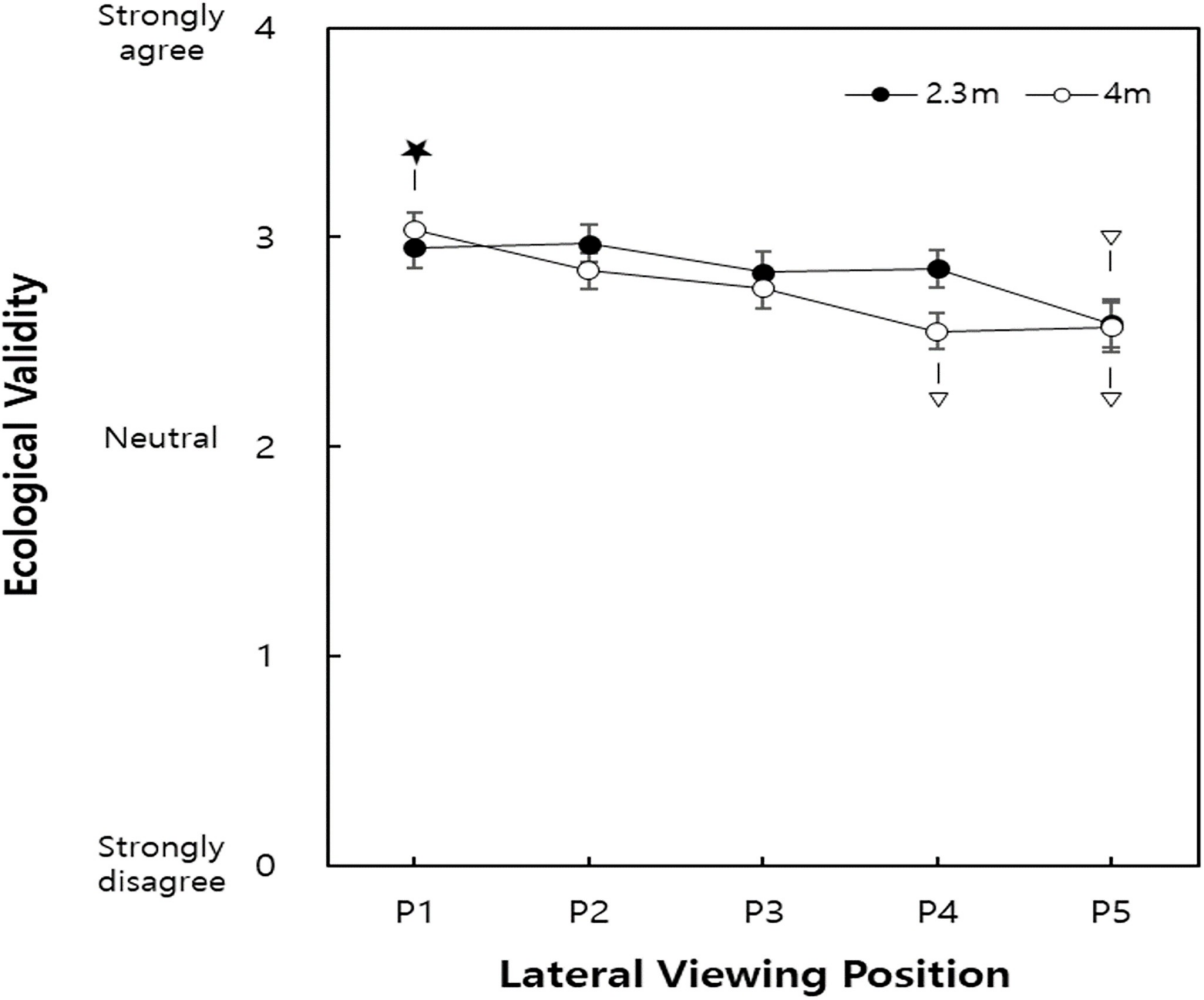
Interactive effects of viewing distance × lateral viewing position on ecological validity (P_1_: Central position, P_5_: Rightmost position (140 cm off-center). Among the treatments belonging to Group A according to Tukey’s HSD test, the treatment with the highest mean ecological validity denoted as ★. Treatment not belonging to Group A denoted as ▽. Treatments without ▽ belong to Group A with the treatment with ★. Range of SEs: 0.08–1.12).

The interaction effect of viewing distance × lateral viewing position was significant for ecological validity (*p* = 0.031). Six of the ten treatments were in the same group (A) with 4m-P_1_ (viewing distance-lateral viewing position), which provided the highest mean (SD) ecological validity of 3.03 (0.62) (Fig 7).

### Effects of display curvature radius

Although display curvature radius (*p* = 0.025) significantly affected negative effects, the five display curvature radius levels were placed in one group according to post hoc testing.

### Effects of viewing distance

**Fig 8:**
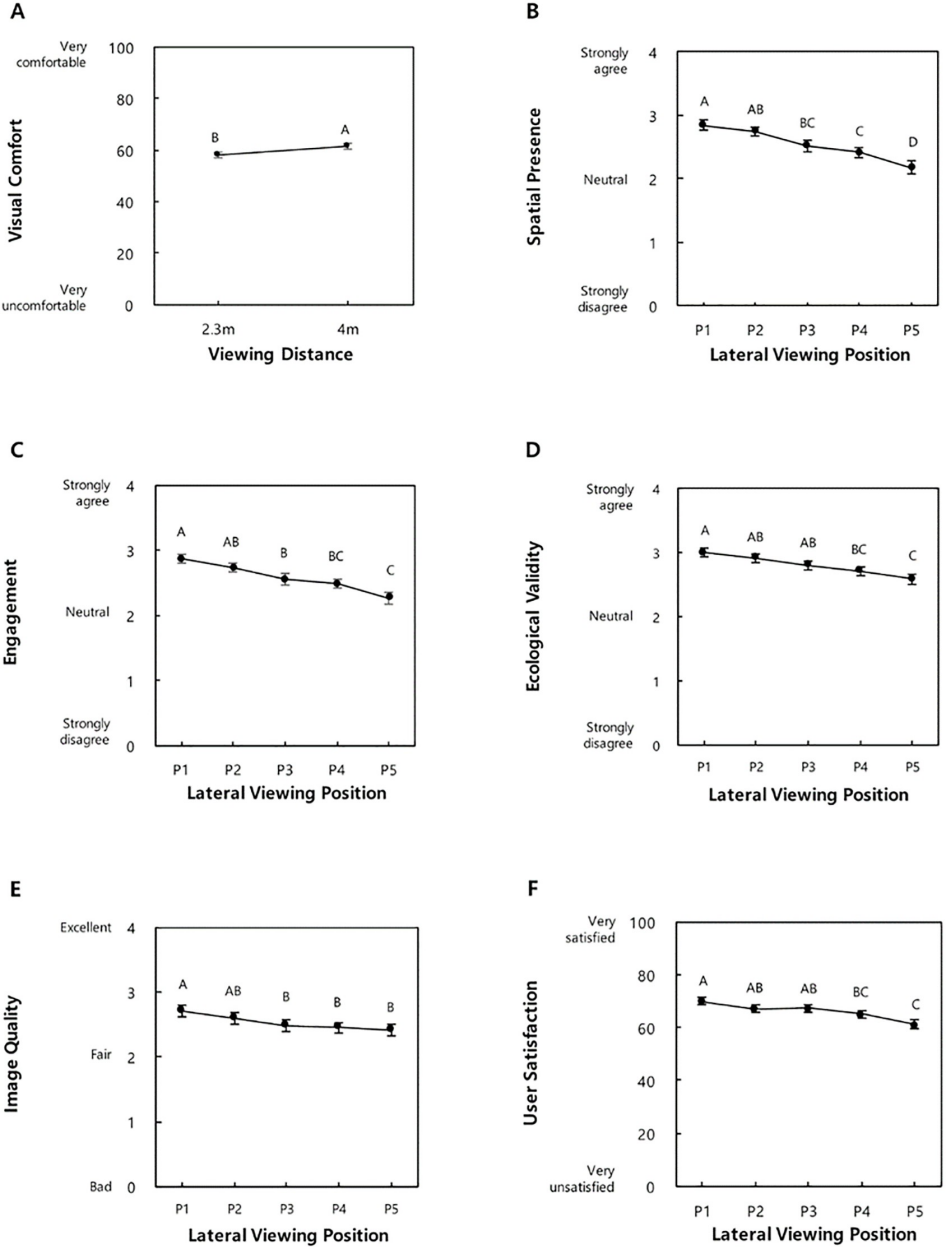
Effect of viewing distance on visual comfort (A), and effects of lateral viewing position on spatial presence (B), engagement (C), ecological validity (D), image quality (E), and user satisfaction (F) (Tukey’s HSD grouping indicated above the mean values; Error bars indicate standard errors).

Viewing distance significantly affected visual comfort (*p* = 0.035) with a higher mean (SD) at a viewing distance of 4 m vs. 2.3 m (61.4 (19.3) vs. 58.1 (19.6); Fig 8A).

### Effects of lateral viewing position

Lateral viewing position significantly affected five TV watching experience elements: spatial presence, engagement, ecological validity, image quality, and user satisfaction (*p* < 0.0001). The five lateral viewing position levels were split into two-to-four groups, and the mean values were highest at P_1_ and lowest at P_5_ for all five TV watching experience elements (Fig 8B–8F).

### Regression of user satisfaction on six TV watching experience elements

A stepwise multiple linear regression model using six TV watching experience elements as predictors accounted for 67% of user satisfaction variability (R^2^_adj_ = 0.67). Multicollinearity was not severe, with variance influence factors (VIF) ranging between 1.2–1.6 (Table 4; [80]). Based on standardized beta weights, the engagement (highest), visual comfort, and image quality were more determinative of user satisfaction than negative effects (lowest; see Table 4). Two TV watching experience elements, spatial presence and ecological validity, were excluded from the final stepwise regression model.

**Table 4:** Regression coefficients, standardized beta weights, and VIFs for the stepwise regression model of user satisfaction using six TV watching experience elements (spatial presence and ecological validity excluded).

Predictor	Coefficient	Standardized beta weight	VIF	p-value
**Intercept**	17.60	0	-	<0.0001
**Engagement**	7.87	0.43	1.3	<0.0001
**Visual comfort**	0.33	0.40	1.6	<0.0001
**Image quality**	3.94	0.22	1.2	<0.0001
**Negative effects**	–1.67	–0.08	1.3	0.006

## Discussion

This study considered three media form factors (display curvature radius, viewing distance, and lateral viewing position) and examined their effects on seven TV watching experience elements. Although display curvature radius alone did not appreciably affect any of the seven TV watching experience elements, the interaction of display curvature radius × viewing distance × lateral viewing position significantly affected both spatial presence and engagement, indicating that the display curvature radius indirectly affected TV watching experience. Indeed, 4000R–4m–P_1_ (display curvature radius-viewing distance-lateral viewing position) exhibited the highest mean spatial presence and engagement. A further analysis recommended that the display curvature radius be equal to the viewing distance and lateral viewing position be P_1_ or P_2_ for a better TV watching experience. Among the three media form factors, lateral viewing position was the most influential to TV watching experience. Among the six TV watching experience elements, engagement was most influential to user satisfaction. Below, each effect is interpreted more in detail, and the effects of the field of view and viewing angle on TV watching experience are additionally discussed, as these two factors vary with the display curvature radius, viewing distance, and lateral viewing position.

### Interaction effects

The interaction of display curvature radius × viewing distance × lateral viewing position significantly affected spatial presence and engagement. Spatial presence increased when the display curvature radius approached a viewing distance, and the lateral viewing position was less off-center, with the highest spatial presence observed at 4000R–4m–P_1_ (display curvature radius-viewing distance-lateral viewing position). Additionally, the lateral viewing position affected spatial presence more adversely for the flat vs. curved screen for both viewing distances. Engagement exhibited similar results to spatial presence, with the highest engagement observed at 4000R–4m–P_1_.

In addition, the interaction of viewing distance × lateral viewing position significantly influenced ecological validity. For viewing distances of 2.3 m and 4 m, ecological validity decreased as the lateral viewing position was more off-center, with a more prominent effect of the lateral viewing position observed at a viewing distance of 4 m. Specifically, ecological validity significantly decreased at P_5_ (140 cm) for a 2.3 m viewing distance vs. P_4_ (109 cm) for a viewing distance of 4 m. Therefore, sitting closer (2.3 m vs. 4 m) could be considered to improve ecological validity, especially when the lateral viewing position is inevitably approximately 1 m off-center (e.g. in multi-viewer conditions).

### Effects of display curvature radius

Display curvature radius alone did not appreciably affect any of the seven TV watching experience elements. Contrarily, some previous studies showed that curved displays provided better TV watching experiences than flat displays. A study [68] found that the visual presence at a viewing distance of 2 m was 18% (for 2D content) and 9% (for 3D content) higher on a 45" 4200R curved TV screen relative to a 45" flat screen, argued to be due to improvements in visual sensitivity at the lateral areas of the curved screen. ‘Realness’ considered as a presence factor during watching was higher on 55" curved TVs relative to their flat counterpart when the viewing distance (5 m) was equal to the display curvature radius. Varying experimental durations and visual stimuli appear to have created these discrepancies [31].

### Effects of viewing distance

**Fig 9:**
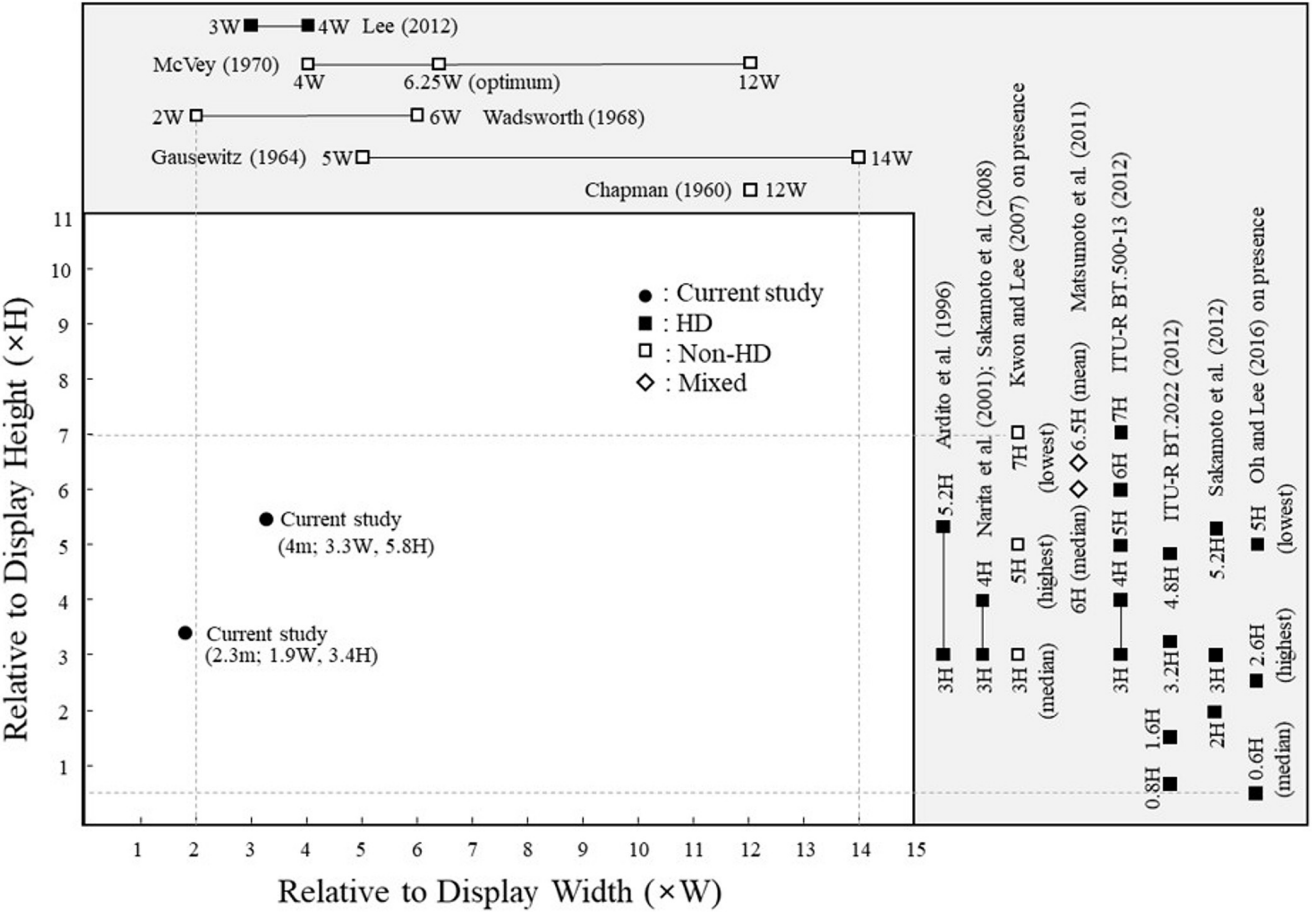
Viewing distances used in the current study vs. those from the literature (Data in the grey area are available only in terms of display height or display width; recommended range values are indicated by solid lines).

In this study, viewing distance was significant only for visual comfort, with 6% greater comfort at 4 m (5.8 H) than 2.3 m (3.4 H). These two viewing distances were within the range recommended (3–7H for flat HD TVs [19]), although the 4 m (5.8 H) viewing distance exceeded the ranges recommended for non-HD TVs, 5H (29”), 3–5.2 H (38"), 3–4 H, and 0.8–4.8H for HD TVs [27, 40, 41, 45, 46], (see Table 5 and Fig 9). As median and mean viewing distances observed in homes of 6 H and 6.5 H [43], respectively, viewing distances outside the above recommended ranges appear common in practice. It was reported that the mean preferred viewing distance for visual comfort using HD TVs was 3.8 W (6.8 H) for 32" TVs, 3.6 W (6.5 H) for 37" TVs, and 3.6 W (6.5 H) for 42" TVs [80]. These values are also above the values (6 H for 36" and 5 H for 73" HD TVs) recommended by ITU [42]. It should be noted that these studies involved different display sizes and resolutions.

**Table 5:** TV viewing distances used in the current study vs. those from the literature.

TV viewing distances		Viewing distance (m)	Relative to display width	Relative to display height	References
**Used in the current study**		**2.3 m** **4 m**	**1.9 W** **3.3 W**	**3.4 H** **5.8 H**	**-**
**Recommended for flat TV**	**Non-HD**^†^ **TV (analog TV)**		12 W (max)		[81]
			5–14 W		[38]
			2–6 W		[39]
			4–12 W, 6.25 W (optimum)		[44]
		2 m 1.3 m 3 m		5 H (highest presence) 3 H (median) 7 H (lowest) on 29"	[40]
	**HD**^†^ **TV**			3–5.2 H (38")	[27]
				3–4 H	[45]
				3–4 H (42" PDP TV)	[46]
			3–4 W (32", 37", and 42")		[82]
	**SD or HD TV**			7 H (27") 6 H (36") 5 H (73") 3–4 H (120")	[42]
				4.8 H (1280×720 pixel) 3.2 H (1920×1080 pixel) 1.6 H (3840×2160 pixel) 0.8 H (7680×4320 pixel)	[41]
		1.1 m (17") 1.7 m (42" & 65")		5.2 H (17") 3 H (42")/2 H (65")	[31]
	**UHD TV**	2 m (2.5H) 0.5 m (0.6H) 4 m (5H)		2.5 H (highest presence) 0.6 H (median) 5 H (lowest) on 65" TV	[68]
**Observed**		3.4 m (mean)			[48]
		2.7 m (mean)			[83]
		2.7 m (mean)			[49]
		2.5 m (median)		6.0 H (median) 6.5 H (mean)	[43]
**Surveyed**		2–3 m (53% of 157 households)			[84]

SD = Standard-Definition; HD = High-Definition; UHD = Ultra High-Definition; PDP = Plasma Display Panel;

^†^Non-HD includes SD.

Although viewing distance had no significant effect on the four sub-concepts of presence investigated here (*0*.*067≤ p ≤ 0*.*29*), three sub-concepts of presence (excluding negative effects) were perceived higher at 2.3 m than 4.0 m. An appropriate viewing distance for a given display size is generally required to enhance presence, whereas watching TV from excessively short or long distances decreases presence [36, 37]. The presence of 29" analog TVs was highest at a viewing distance of 5 H (2 m), followed by 3 H (1.3 m) and 7 H (3 m) [40]. It was found that involvement [31], similar to engagement [62], was highest at a viewing distance of 5.2 H (1.1 m) for 17" TVs, 3 H (1.65 m) for 42" TVs, and 2 H (1.65 m) for 65" TVs, respectively. Additionally, it was found that a viewing distance of 2.5 H (2 m) provided the highest visual presence when watching 2D images on 65" flat ultra-high-definition (UHD) TVs, followed by 0.6 H (0.5 m) and 5 H (4 m) [68]. When similar viewing conditions are considered, the current results resemble those of these studies [31, 40, 68].

### Effects of lateral viewing position

This study recommends viewing positions P_1_ and P_2_ (or a lateral viewing position < 70 cm off-center) for watching TV. More off-center positions than P_2_ degraded TV watching experience, evidenced by decreases in the spatial presence (11–23% for P_3_–P_5_), engagement (11–21% for P_3_–P_5_), ecological validity (10–24% for P_4_–P_5_), image quality (9–11% for P_3_–P_5_), and user satisfaction (7–12% for P_4_–P_5_) relative to P_1_. Such degradations can be attributed to the decrease in the field of view and increase in the viewing angle caused by more lateral deviations of the viewing position.

### Effects of field of view and viewing angle

In this study, the field of view did not appear to influence TV watching experience substantially. Geometrically, the field of view increases as the display curvature radius approaches a viewing distance, a viewing distance decreases, or a lateral viewing position approaches the central position. In addition to shorter viewing distances [85] and larger display sizes [85], higher attention and arousal levels due to a wider field of view [86] can increase presence, a feeling of being in a virtual world. Presence is influenced by the relative amount of information incoming from the virtual compared with the physical environment [87]. Presence increases if the viewer’s visual field of view is more occupied by the on-screen image [88]. Though the magnitude of the field of view was predominantly determined by viewing distance in this study, the effects of viewing distance (2.3 m and 4 m) on spatial presence, engagement, and ecological validity were non-significant. A wider field of view at a viewing distance of 2.3 m did not significantly increase presence, presumably due to the decrease in visual comfort created by the shorter viewing distance (visual comfort at 2.3 m was 5.4% lower than at 4 m). Conversely, lateral viewing position significantly affected presence, although it affected the field of view less than viewing distance. Fields of view at 2.3 m were wider than those at 4 m by up to 12.8° across lateral viewing positions, whereas the difference in the fields of view between viewing distance 2.3 m and 4.0 m at the same lateral viewing position was ≤ 8° (See Table 3). Some prior studies using varying screen sizes rather than viewing distances showed that the field of view significantly influenced presence. It was found that the physical presence during a 30 min gaming task was higher on an 81" screen (diagonal field of view = 76°) than on a 13" screen (18°) [67]. The perceived presence during a driving task on a triple screen comprising three 2300 × 1750 mm screens was highest with a 180° field of view, followed by 140° and 60° [89]. However, the effect of change in viewing angle (as determined by lateral viewing position) on presence was not examined in these two studies.

In the present study, presence decreased as viewing angles increased (or lateral viewing positions were more off-centered). Specifically, significant decreases in presence (in terms of spatial presence, engagement, and ecological validity) began at a viewing angle of 17.0° (P_3_) for a 2.3 m viewing distance and 9.9° (P_3_) for a 4 m viewing distance. Previous studies reported mixed results. In one study, the visual presence of a 2D image on a 65" UHD flat TV at a viewing distance of 2 m deceased by 17% when the viewing angle was increased from 0° to 45° [54]. Conversely, the presence on an 86" screen at a viewing distance of 0.9 H (1.75 m) did not significantly change at three viewing angles (–19°, 0°, and +19°) [16]. This inconsistency is presumably due to the increased presence with the combined effect of a larger screen size (55–86") and a closer viewing distance (2–1.75 m) vs. 55"-2.3 m or 65"-2 m.

In the current study, image quality decreased as viewing angles increased (or lateral viewing positions were more off-centered). Significant decreases in image quality began at a viewing angle of 17.0° (P_3_) for a 2.3 m viewing distance and 9.9° (P_3_) for a 4 m viewing distance. Previous studies reported similar results. The quality of 2D images on 55" flat and curved TVs at a viewing distance of 2.2 m was degraded as viewing angle increased from 0° to 30°, with a more severe degradation observed with a flat TV [55]. Similarly, the quality of 2D images on flat displays at a 6H viewing distance decreased as viewing angle increased from 0° to approximately 80° [71].

Decreases in presence and image quality with increasing viewing angle (or at more off-center lateral viewing positions) observed in the current study appear to be in part due to the perceived image distortion with the increase in viewing angle [6]. In the current study, image quality at P_1_ and P_2_ was comparable across the four different display curvature radii. Viewing angles at a viewing distance of 2.3 m and at P_3_ increased by up to 29.6° for a flat TV, and image quality began to degrade. These results were in accordance with a previous finding—perceptual constancy observed within viewing angles ≤ 28.6° [9]. In addition, the image quality was positively correlated with the three sub-concepts of presence, namely, spatial presence, engagement, and ecological validity, with bivariate correlations of 0.40, 0.36, and 0.53 (*p* < 0.0001), respectively.

To better examine the effect of an actual TV viewing context on TV watching experience, it seems necessary to allow for wider viewing angles. Although the largest viewing angle considered in this study (30.3° at a viewing distance of 2.3 m) exceeded the mean viewing angle of 23.3° obtained in a field survey [48], viewing angles observed in actual households have ranged between ± 30° [90], ± 45° [91], and ±60° [49]. Of note, however, the current study recommends P_1_ or P_2_ (or lateral viewing positions closer than P_3_) for a better TV watching experience, and the viewing angle for 2.3m-P_3_ was 17°.

### Regression of user satisfaction on six TV watching experience elements

In the current study, a regression model (R^2^_adj_ = 0.67) for user satisfaction was developed using six TV watching experience elements. Based on the standardized beta weights, engagement, visual comfort, and image quality were 5.4 times (=0.43/0.08), 5.0 times (=0.40/0.08), and 2.8 times (=0.22/0.08) more influential to user satisfaction than negative effects, indicating that improving these three TV watching experience elements can improve user satisfaction more effectively. Engagement increased when the display curvature radius was equal to the viewing distance and the lateral viewing position was < 70 cm off-center (Figs 6 and 8C). The mean visual comfort rating was higher with a viewing distance of 4 m (Fig 8A). The image quality increased when the lateral viewing position was < 70 cm off-center (Fig 8E). Therefore, 4000R-4m-P_1/2_ (display curvature radius-viewing distance-lateral viewing position) is recommended for user satisfaction.

### Limitations and future studies

Some limitations were encountered in the current study. First, display curvature radii were simulated using projection films and a beam projector instead of actual display panels. Although comparatively high-fidelity mock-ups were used in this study (vs. static images attached to curved surfaces [6, 11]), these mock-ups were different from actual displays. Second, 5 min videos were used in experiments. Previous studies on presence used task durations ranging from 1.5 min [69] to 1 h [31]. An additional study is warranted to examine the effects of display curvature radius, viewing distance, and lateral viewing position on diverse TV watching experience elements during longer-term TV watching. Third, subjective ratings were used to assess TV watching experience. Some behavioral or physiological measures are available to assess presence, visual comfort, image quality, and user satisfaction (including eye movements [92], electrocardiograms [92], and electroencephalograms [93]). Additional studies are necessary to develop validated objective measures and experimental methods that can account for the TV watching experience of multiple viewers simultaneously and to support conclusions of this study drawn based on subjective measures only. In addition, it would have been better to obtain a simultaneous judgment of confidence for each subjective rating made by the participant. Fourth, the effects of gender, age, and personal characteristics were not considered. The effect of display size on presence was not significant in the male group, whereas the female group reported higher presence with wider displays [1]. A separate study [40] revealed that those with higher immersive tendencies reported higher presence during TV watching, but observed no significant gender effects. TV watching experience could also be affected by ocular changes with age (e.g. functional degradations of the visual system with age [94] and visual fatigue in presbyopic eyes [95]). Personal characteristics (such as a willingness to suspend disbelief, knowledge or prior experience with the medium, and personal types [96]) are also important factors for presence. Fifth, in addition to the three media form factors (display curvature radius, viewing distance, and lateral viewing position) considered in this study, media content factors (overall theme, narrative, and story) can influence TV watching experience in terms of involvement [72], engagement, and ecological validity [73]. To examine the effects of the three media form factors on TV watching experience, this study controlled media content factors using similar videos. Finally, this study considered 55" screen sizes and two viewing distances; other screen sizes and viewing distances should be considered in future studies. Despite the above limitations, the findings of this study can help determine effective combinations of display curvature radius, viewing distance, and lateral viewing position for a better TV watching experience with 55" TVs.

## Conclusions

The current study examined the effects of display curvature radius, viewing distance, and lateral viewing position on TV watching experience. The interaction effect of display curvature radius × viewing distance × lateral viewing position was significant for spatial presence and engagement. Spatial presence and engagement increased when the display curvature radius approached a viewing distance and a lateral viewing position was less off-center. However, display curvature radius alone did not appreciably affect TV watching experience, and viewing distance alone significantly affected visual comfort only. Overall, lateral viewing position was the most influential on TV watching experience. With increasing lateral viewing position, spatial presence, engagement, ecological validity, image quality, and user satisfaction decreased. Lateral viewing position < 70 cm is recommended for a better TV watching experience. Among the six TV watching experience elements (spatial presence, engagement, ecological validity, negative effects, visual comfort, image quality), engagement accounted for the highest degree of user satisfaction. These findings can contribute to enhancing TV watching experience by specifying effective combinations of display curvature radius, viewing distance, and lateral viewing position as well as by manifesting the relative importance of each TV watching experience element in explaining user satisfaction.

## Supporting information

S1 Data (XLSX)
